# Anti-DEFA5 Monoclonal Antibody Clones 1A8 and 4F5 Immunoreactive Bioassay for Diagnosing Inflammatory Bowel Disease

**DOI:** 10.21203/rs.3.rs-4843765/v1

**Published:** 2024-08-29

**Authors:** Rabi Thangaiyan, Amos M. Sakwe, Alexander T. Hawkins, Mary K. Washington, Billy R. Ballard, Michael G. Izban, Sanika S. Chirwa, James E.K. Hildreth, Anil Shanker, David L. Blum, Amosy E. M’Koma

**Affiliations:** Meharry Medical College; Meharry Medical College; Vanderbilt University Medical Center; Vanderbilt University Medical Center; Meharry Medical College; Meharry Medical College; Meharry Medical College; Meharry Medical College; Meharry Medical College; University of Georgia; Meharry Medical College

**Keywords:** IBD, DEFA5, 1A8, 4F5, specificity

## Abstract

**Background:**

Robust evidence suggests that the aberrant expression of α defensin 5 protein (DEFA5) in colon inflammatory bowel diseases (IBDs) underlies the distinct pathogenesis of Crohn’s colitis, can be exploited as a reliable diagnostic biomarker to differential diagnosis of Crohn’s colitis (CC) from Ulcerative colitis (UC) in otherwise indeterminate colitis (IC). We evaluated the specificity of the commercially available anti-DEFA5 antibodies and showed further validation of their appropriateness for a given application is required.

**Methods:**

We established two mouse monoclonal DEFA5 antibody clones 1A8 and 4F5 by immunizing the mice with purified recombinant protein and validated the specificity, selectivity and cross reactivity in recognizing the endogenous and recombinant DEFA5 protein, especially for Immunohistochemistry, Western blot, Immunoprecipitation, or enzyme-linked immunosorbent assay.

**Results:**

Clones 1A8 and 4F5 recognized effectively the endogenous DEFA5 in active human diverticulitis (DV), UC, CC or IC disease samples, including transiently transfected HEK293T cells expressing DEFA5 with high degree of specificity and minimal non-confounding cross reactivity.

**Conclusions:**

1A8 and 4F5 clones are worth studying in larger IBD cohorts to fully address whether DEFA5 expression may be used as a diagnostic biomarker to discrimination of the diagnosis of UC from CC or IC into authentic CC or UC or a colitis with different pathological characteristics.

## Introduction

Antibodies are commonly used to validate targets and are powerful tools in western blotting (WB), immunohistochemistry (IHC), immunoprecipitation (IP), Enzyme-Linked Immunosorbent Assays (ELISA), and Flow Cytometry applications. They are also vital tools in clinical management, with extensive use in a large number of clinical laboratory assays (ELISA and flow cytometry) and diagnostic anatomic pathology practice. As monoclonal antibodies possess high target specificity, we established hybridoma cell lines that stably secrete anti-DEFA5 antibodies as a powerful tool in this study. Inflammatory bowel disease (IBD) which includes ulcerative colitis (UC) and Crohn’s colitis (CC) affects millions of people in the United States and its incidence and prevalence is increasing worldwide and has become a global emergent disease [1, 2]. Colonic IBD is a highly heterogeneous, chronic, relapsing, and remitting disorders [2–6]. Failure to differentiate between the diagnostic features of predominantly colonic IBD, that is, UC and CC, may lead to an inexact diagnosis of indeterminate colitis (IC). The diagnostic accuracy of indeterminate colitis in either UC or CC in otherwise IC patients is of utmost importance when determining a patient’s candidacy for pouch surgery and restorative proctocolectomy with ileal pouch-anal anastomosis (RPC-IPAA) due to pharmaceutical refractoriness [7]. Furthermore, incorrect diagnosis and treatment can result in potential morbidity owing to inappropriate and unnecessary surgery. Over 15% of adult patients with colonic IBD continue to experience diagnostic ambiguity and delay [7], and 20% of pediatric IBD cases occur within three years of symptom onset and IBD diagnosis [8, 9]. Our published data has shown robust evidence supporting the presence of alpha defensin 5 (DEFA5 alias HD5) in areas of the colon mucosa with aberrant expression of apparent Paneth cell-like cells (PCLCs) or crypt cell-like cells (CCLCs), which identifies an area of **colonic ileal metaplasia** consistent with the diagnosis of CC [10, 11]. DEFA5 immunoreactivity in IC patient samples facilitates CC diagnosis, with a positive predictive value (PPV) of 96% [7]. We also observed a fit logistic model with group CC vs. UC as the outcome and DEFA5 as the independent biosignature discriminator for patients with IBD (10). CCLCs and DEFA5 are colocalized, which reconciles and represents a consensus among treating physicians [7, 10]. Human endoscopic terminal ileal fluid analysis confirmed the predominant *in vivo* form of mature DEFA5 (amino acids 63–94) and slightly longer (amino acids 56–94) predicted by *in vitro* trypsin cleavage [12]. Therefore, it has been proposed that enzymatically active trypsin converts pro-DEFA5 to its mature form *in vivo* [13, 14]. Human enteric α-defensins DEFA5 and DEFA6 have been identified and shown to be predominantly expressed in the Paneth cells of the small intestine, as determined by in situ hybridization [15, 16]. It has been reported that DEFA5 has been isolated from ileal tissue and characterized [17, 18]. Recombinant DEFA5 has been produced [19, 20] and shown to have a wide spectrum of bactericidal effects against bacteria and fungi [20, 21]. Four human α-defensins, myeloid defensins HNP-1, HNP-2, HNP-3, and HNP-4, are expressed in neutrophil cells [22] and exhibit a broad range of antimicrobial activities [23].

Due to the sequence homology of the α-defensin class of proteins (DEFAs, 1–6), antibody assays recognize both DEFAs, active DEFA5 and inactive pro-DEFA5 [1, 2]. It is necessary to develop antibody pairs (capture and detection) and purified recombinant antigens/proteins to determine the specificity and sensitivity of DEFA5 to attain the maximum signal-to-noise ratio, which is diagnostic for the IBD subtype and NOT other DEFAs. This constitutes the development of a point-of-care, rapid and accurate diagnosis of IBD using DEFA5 bioassay that potentially qualifies as FDA defined “Diagnostic” “Prognostic” and Monitoring” biomarker(s) [PMID: 22729569]. Widespread use of a DEFA5 bioassay would facilitate not only instant accurate diagnosis and eliminate diagnostic ambiguity as IC in colonic IBD, but also circumvent diagnostic delay, prompting timely appropriate treatment regimens and improved health outcomes and quality of life, while reducing medical complications, unnecessary surgery, and health care costs. Therefore, the development of molecular tools that allow the detection of DEFA5 to distinguish IBD subtypes is of primary importance. To achieve this, we developed two in-house clones 1A8 and 4F5 targeting DEFA5 monoclonal antibodies (mAbs), and two U.S. patents that cover the protein compounds, compositions, and mAbs potentially more selective and avid against DEFA5 for exact binding to produce adequate specificity, which only recognizes DEFA5, which is diagnostic for the IBD subtype. CC patents have been granted by the United States Patent and Trademark Office (USPTO) [24, 25]. In this study, we validated their specificity in recognizing endogenously expressed DEFA5 through Western blotting, immunohistochemistry, immunoprecipitation of multiple epitope-specific antibodies that recognize distinct epitopes, and Sandwich ELISA.

## Materials and Methods

### Cloning, expression and purification of recombinant DEFA5 protein

The DEFA5 gene was codon-optimized for expression in *Escherichia coli* and was custom-synthesized by GenScript company. DEFA5 was cloned using two PCR primers containing Nidel and HindIII restriction enzyme digestion sites. The PCR product was ligated to the vector pET30a with a His tag, and the ligated product was transformed into *E. coli* BL21 Star^™^ (DE3). The recombinant protein was identified using restriction enzyme digestion and PCR amplification. The positive recombinant transformants were inoculated into LB medium containing 50 μg/ml kanamycin, incubated with shaking at 220 r/min at 37°C for 6 h, and then induced with 1 mM IPTG at 37°C for 4 h and 15°C for 16 h. SDS-PAGE was used to monitor the expression. Cells incubated at 15°C for 16 h were harvested at O. D. 1.2, centrifugation at 12000 rpm for 2 min, and total cellular pellets were analyzed by SDS-PAGE and Western blotting. Cell pellets were resuspended in lysis buffer and sonicated. After centrifugation, the supernatant was collected. The target DEFA5 protein was obtained by two-step purification using a Ni column and Superdex 75 column, and the protein was sterilized a 0.22 μm filter before being stored in aliquots at −80°C. The protein concentration was determined using the BCA protein assay, with BSA as the standard. Protein purity and molecular weight were determined by standard SDS-PAGE and western blotting, respectively.

### Immunization and production of hybridomas

Five BALB/c mice were immunized with 10 μg of purified recombinant protein per mouse in Freund’s adjuvant, and after two weeks, the mice were boosted with the same protein. Blood samples were collected from the tail vein two weeks after the second immunization to measure serum antibodies. Blood serum was separated and subjected to Enzyme-Linked Immunosorbent Assay (ELISA) to determine the antibody titer. Serum from non-immunized mice was used as the control. The mice were then boosted with the same immunogen, and the highest titer mouse was used for fusion after five immunizations. The mice were euthanized, their spleens were removed, and spleen cells were prepared. Single immune spleen cells were fused with SP2/O myeloma cells (ratio 1:1) by co-centrifugation in polyethylene glycol. Myeloma cells were prepared by growing 8-azaquanine for one week before cell fusion. Cells were cultured in 96-well plates in 20% RPMI-1640 medium with hypoxanthine-aminopterin-thymidine (HAT) used with hybridoma selection. Hybridoma cultures were screened for antibody production using ELISA and positive hybridomas were cloned.

### Enzyme-Linked Immunosorbent Assay

ELISA was used to screen for the induction of antibody responses in the sera of immunized mice for the selection of antigen-specific hybridoma cell lines and mAb isotype determination. Briefly, ELISA plates were coated with 10 ng/mL purified DEFA5 protein in a carbonate/bicarbonate coating buffer. The plates were incubated overnight at 4°C, washed twice with wash buffer, and saturated with blocking buffer (2% BSA in PBST) for 2 h at room temperature. Serum samples were added to PFT buffer (PBS containing 0.05% Tween 20) after serial dilution from 1:50 to 1:10000 and hybridoma and clone cell supernatants were diluted 1:2. After incubation for 2 h, the plates were washed with wash buffer and incubated for 1 h with the Peroxidase AffiniPure Goat Anti-Mouse IgG (H + L) antibody (Fisher Scientific). After washing three times, the TMB substrate solution was added, followed by the addition of 2M sulfuric acid to stop the reaction. Absorbance was measured using a microplate reader at 45m nM. Sera from unimmunized mice were used as negative controls.

### Cloning of hybridomas

The strongest hybridoma pools were subcloned using standard limiting dilution to generate monoclonal hybridoma cell lines. Hybridomas 1A8 and 4F5 were expanded, and the medium was clarified by centrifugation to remove cells and then passed over a Protein A column to bind the mAb. The resulting mAbs ( 1A8 and 4F5) were eluted in glycine pH2.5, dialyzed into PBS, sealed into tubes, and stored at −80°C after validation by SDS-PAGE. Isotyping of clone 1A8 and 4F5 mAbs was performed using isotyping enzyme-linked immunosorbent assay (ELISA) kits (Invitrogen).

### Immunohistochemical staining

Five-micron sections of the formalin-fixed paraffin-embedded normal terminal ileum were deparaffinized in xylene, rehydrated using graded alcohols, and finally in water. Heat-induced epitope retrieval was carried out using the Labvision PT module and PT module 1 mM EDTA (pH 8.0) containing 0.05% Tween-20 (Thermo Scientific, Waltham, MA) at 98°C (60°C preheat/70°C cool down) for 20 min. Immunostaining was performed on a LabVision Autostainer using an Ultravision Quanto (HRP polymer) Detection System (Thermo Scientific). Standard incubation times were used, except that the primary antibody was incubated for 60 min followed by a stringent 5-min wash in TBS containing 0.1% Tween-20. Antibodies 1A8, 4F5, and R&D972207 were used at a 1:50 dilution in OP Quanto antibody diluent (Thermo Fisher, Waltham, MA). The Quanto DAB Plus system was used for 5 min for color development. The slides were counterstained for 1 min with Mayer’s hematoxylin, dehydrated, and coverslipped using Cytoseal XYL (Thermo Scientific, Waltham, MA). Images were captured using a Nikon Eclipse E400 microscope equipped with a Motic 5 MP digital webcam.

### Preparation of tissue lysates and Western blot analysis of DEFA5

Frozen tissue samples from patients with diverticulitis (DV), such as non-IBD control, ulcerative colitis (UC), Crohn’s colitis (CC), and indeterminate colitis (IC), were washed with chilled 1x PBS to remove blood and minced into small pieces on ice. Tissues were homogenized in 10 volumes of ice-cold RIPA buffer containing a cocktail of protease inhibitors and sonicated for 10 seconds/10 seconds at rest for each cycle for 90 s using an ultrasonic homogenizer. Homogenates were centrifuged at 14,000 rpm at 4°C for 20 min to pellet cell debris, and the supernatant was transferred to a fresh microfuge tube. Protein concentration in the tissue lysates was determined using the BCA protein assay. For Western blotting, equal amounts (30 μg) of protein from the tissue lysates were mixed with 2x SDS sample buffer (4% SDS, 125 mM Tris-HCl pH 6.8, 10% glycerol, 100 mM DTT, 0.002% bromophenol blue), incubated at room temperature for 1 h, loaded on a 12% NuPAGE gel (1.5 M Tris-pH 8.8, 30% acrylamide bis, 10% AP, and TEMED), and run in Tris/glycin/SDS electrophoresis buffer. The proteins were transferred to a PVDF membrane using a mini trans-blot cell at 300 mA in transfer buffer (25 mM Tris pH8.3, 192 mM glycine, and 20% methanol) at 4°C for 1.5 h and the membranes were blocked for 1 h at room temperature (5% dry milk, 0.5% Tween-20, PBS). The membranes were then incubated with primary antibody (1:500) overnight at 4°C, followed by incubation with anti-mouse IgG peroxidase-conjugated antibody (goat anti-mouse 1:5000, A4416, MilliporeSigma) for 1 h at room temperature. After washing with PBST (3 × 5 min), the signal from the chemiluminescence substrate (Immobilon Western Chemiluminescence HRP Substrate, Millipore) was detected using a ChemiDoc MP imaging system. After detection, the membranes were stripped using a stripping buffer (Invitrogen) and blocked for 1 h. The membranes were incubated with anti-GAPDH antibody (1:10000, G9545, Millipore Sigma) for 1 h at room temperature, followed by incubation with anti-rabbit IgG peroxidase antibody (goat anti-rabbit, 1:5000, A0545, Millipore Sigma) for 1 h, and the signal was detected as described above.

### Identification of DEFA5 capture antibodies by Immunoprecipitation/Western blotting

Our antibodies (clones 1A8 and 4F5) and commercially available anti-DEFA5 antibodies (sc-53997 and R&D972204) were used to immunoprecipitate endogenous DEFA5 from CC tissue lysates. For this assay, 1 mg of protein in 1000 μl lysis buffer was mixed with 20 μl of 50% Protein A/G bead slurry prepared in 0.1% BSA in PBS and incubated with end-over-end rotation at 4°C for 2 h, then centrifuged at 14,000 × g for 10 min at 4°C and the pre-cleared supernatant was transferred to fresh tubes. The indicated antibodies were added to 500 μg of pre-cleared protein extract in 500 μl and incubated overnight at 4°C with end-over-end rocking, followed by the addition of 20 μl of 50% Protein A/G beads. These reactions were incubated with end-over-end rocking for 2 h at 4°C, centrifuged at 14,000 × g for 5 min, and the pellet washed three times with wash buffer. The pellets were resuspended in 60 μl 2x SDS sample buffer, boiled for 5 min, and centrifuged at 14,000 × g for 2 min. For standard western blotting, 30 μl immunoprecipitate was loaded onto a 12% SDS-PAGE gel. After gel electrophoresis, the proteins were transferred to PVDF membranes and immunoblotted with R&D972207, 1A8, 4F5, and sc-53997 antibodies, as described above.

#### Assessment of the relative abundance of DEFA5 in control and IBD patient tissues

Based on the IP/WB assay above, we used 1 mg of lysates from DV, UC, CC, and IC patient tissues for immunoprecipitation using the sc-53997 antibody as the capture antibody, followed by western blot analysis as described above. Mouse isotype control IgG was used to confirm the specificity of the capture and detection of DEFA5.

### Immunoprecipitation/Western blots to verify specificity and cross-reactivity of 1A8 and 4F5 antibodies

To determine whether the new antibodies 1A8 and 4F5 were specific for the target protein DEFA5 and did not recognize other DEFA family members, particularly Paneth cell-derived DEFA6, we used purified recombinant DEFA1, DEFA5, and DEFA6 proteins. Recombinant DEFA1, DEFA5, and DEFA6 proteins were immunoprecipitated with sc-53997 antibody or mouse IgG antibody as a negative control. The immune complexes were then subjected to western blotting, as described above.

### Detection of DEFA5 in IBD patient tissues by Sandwich ELISA

To quantitatively determine the DEFA5 protein levels in tissue lysates using 1A8, 4F5, and 972207 antibodies, we developed a Sandwich ELISA procedure. The 1A8, 4F5, and 972207 antibodies were biotinylated using the Abcam 2017960 biotinylation kit, according to the manufacturer’s instructions. ELISA plate wells were coated with 100 μl of sc-53997 capture antibody at 2.5 μg/ml concentration in coating buffer and incubated overnight at 4°C. After washing thrice with wash buffer, 200 μl of blocking solution was added to each well, incubated for 60 min at 37°C and washed thrice with wash buffer. We then added 100 μl of tissue lysates (20 μg/ml) prepared from DV, UC, CC, and IC samples. Standards were serially diluted with the sample diluent and 100 μL was added to each well. The blank wells contained only 100 μL of sample diluent. The plate was incubated at 4°C overnight and, washed in wash buffer, and then 100 μl of biotin-conjugated 1A8, 4F5, and R&D #972207 antibodies was added to each well and incubated for 2 h at 37°C. After washing three times with wash buffer, 100 μl of streptavidin-HRP conjugate was added to each well and incubated for 1 h. Following a wash step to remove any unbound streptavidin-HRP reagent, 100 μl TMB substrate solution was added to each well and incubated for 30 min or until optimal color development was achieved. This was followed by the addition of 100 μl 650 nM stop solution to each well to stop the TMB substrate reaction, and the OD value of each well was immediately measured using a microplate reader at 650 nM.

### Statistical analysis

Statistical analyses were performed using GraphPad Prism version 9. Differentiation in DEFA5 concentrations mean between DV, UC, CC, and IC tissue homogenates was compared using the Student’s t-test. Statistical significance was set at P < 0.05.

## Results

### Expression, purification of recombinant DEFA5 protein and generation of hybridoma cell lines

**Figure 1:**
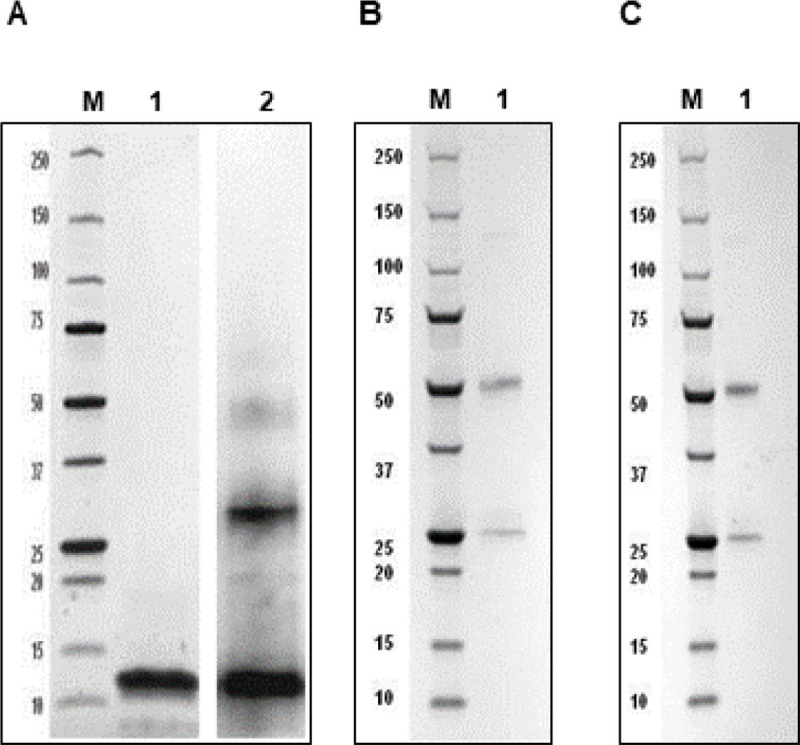
SDS-PAGE analysis of purified recombinant DEFA5 and clones 1A8 and 4F5 mAbs. **(A)**Lane M: Protein marker, Lane 1: DEFA5; Lane 2: DEFA5 reacted with His tag antibody. **(B)** Lane M: Protein marker, Lane 1: 1A8 mAb. **(C)** Lane M: Protein marker, Lane 1: 4F5 mAb.

**Figure 2:**
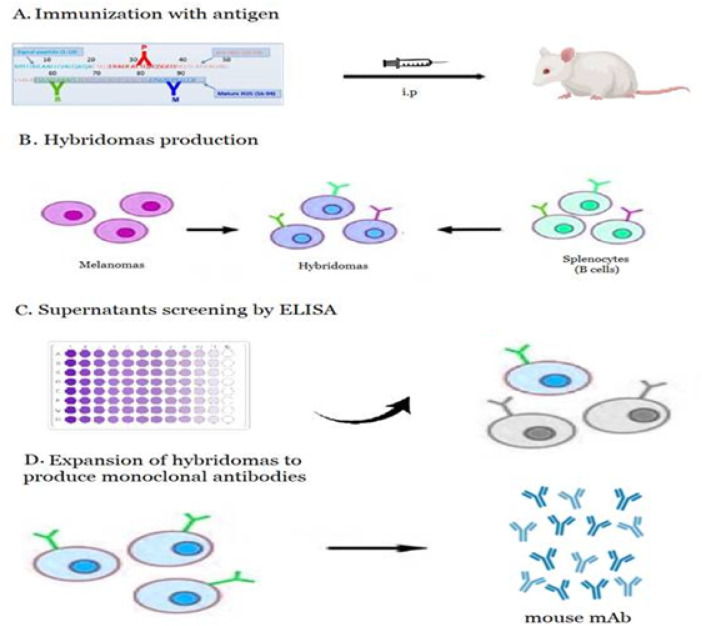
Production of clones 1A8 and 4F5 anti-DEFA5 mAbs. **(A)** Recombinat protein was immunized into Balb/c mice. **(B)** The spleen cells were fused with myeloma cells. **(C)** The supernatants were screened by ELISA to select anti-DEFA5 mAbs producing hybridomas. **(D)** Clones 1A8 and 4F5 anti-DEFA5 mAbs were established after limiting dilution.

The synthetic gene encoding DEFA5 was successfully constructed with a size of 621 bp and inserted into pET30a. The recombinant plasmid was verified by DNA sequencing and restriction enzyme digestion. The prokaryotic expression plasmid, pET30a-DEFA5, was transformed into *E.coli* BL21 Star^™^. The bacterial lysate was centrifuged and the supernatant was loaded onto an affinity column to obtain the purified target protein. The purity of the DEFA5 protein obtained using *E.coli* system was analyzed by SDS-PAGE and was found to be > 90%, with an apparent molecular weight of 12 kDa, which was consistent with the prediction (Fig. 1A). Western blot analysis showed that the expressed DEFA5 protein reacted with the antibody, indicating its biological activity (Fig. 1A). SDS-PAGE analysis demonstrated that the two affinity-purified anti-DEFA5 1A8 and 4F5 mAbs showed a heavy chain band at 50 kDa and a light chain band at 25 kDa, respectively (Fig. 1B and Fig. 1C). To develop anti DEFA5 1A8 and 4F5 mAbs healthy Balb/c mice were immunized with purified DEFA5 (Fig. 2A). The blood sera of the mice were evaluated by ELISA to determine anti DEFA5 antibody titers. The ELISA results indicated that recombinant DEFA5, as an immune antigen, successfully induced the generation of higher antibody titers. Spleen cells harvested from immunized mice with the highest serum titer were fused with Sp2/0 myeloma cells and two hybrid cell lines against DEFA5 were successfully obtained (Fig. 2B). Supernatants from positive cells were tested for antibody production using an indirect ELISA (Fig. 2C) (Supplementary Table 1). After primary and secondary cloning (Fig. 2D), two clones that produced large amounts of antibodies were selected and designated 1A8 and 4F5. Isotyping results showed that the subtype of both mAbs was IgG1, containing *k* light chains.

### Identification of clones 1A8 and 4F5 effective for immunohistochemistry

**Figure 3:**
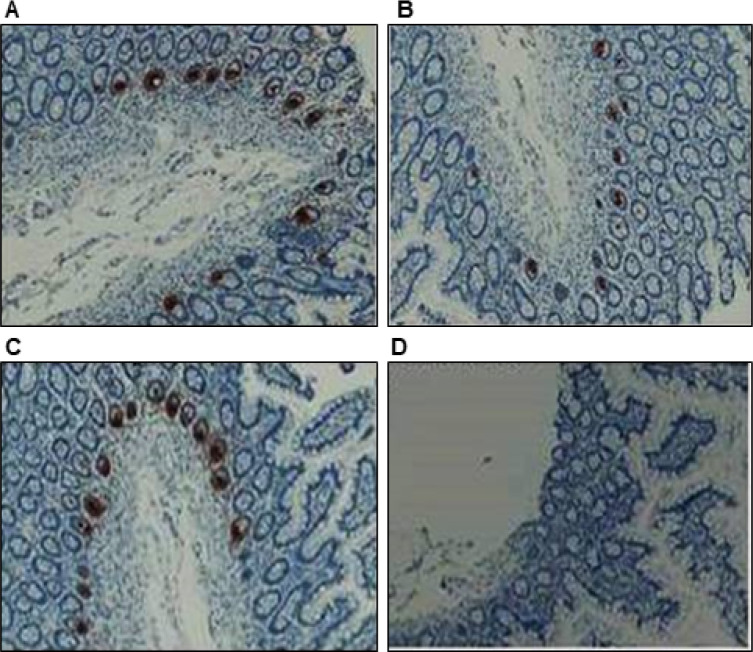
Detection of DEFA5 in sequential sections of normal ileum by Immunohistochemistry assay. Thin sections of normal ileum were stained with two newly developed high-affinity anti-DEFA5 mAbs clones 1A8 and 4F5 compared to the commercially available Santa Cruz Biotechnology Inc antibody (sc-53997), showing ileal paneth cells immunoreactive for 1A8 (**A)**; 4F5 **(B)**; sc-53997 (positive control) **(C)**; and negative control **(D)**.

We used two DEFA5 antibody clones, 1A8 and 4F5, directed against different epitopes of DEFA5 and sc-53997 antibodies to stain serial sections of normal ileum tissue to produce similar results. All three antibodies produced a similar staining pattern in the same tissue and exhibited strong immunoreactivity for DEFA5 in the Paneth cells in a fine granular pattern (Fig. 3A–C), which was abolished in the negative control in the normal ileum (Fig. 3D). These DEFA5 positive cells were also observed in the crypt lumen. Thus, we identified two mouse monoclonal antibodies that effectively and specifically recognized DEFA5 using immunohistochemistry.

### Western blot analysis

**Figure 4:**
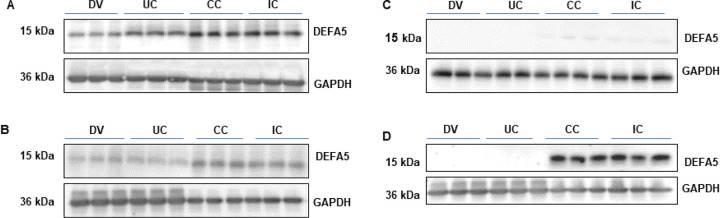
Detection of human α-defensin 5 in tissues from DV control and IDB tissue samples. **(A and B)** DEFA5 expression was detected by Western blotting using our novel DEFA5 antibodies 1A8 and 4F5 and **(C and D)** commercially available DEFA5 antibodies from sc-53997 and R&D972207.. Protein extracts were prepared from tissues obtained from patients with Diverticulitis, a non-inflammatory bowel disease control (DV), Ulcerative colitis (UC), Crohn’s colitis (CC) and Indeterminate colitis (IC).

By using the optimized Western blot protocol as outlined above, our new mouse monoclonal 1A8 and 4F5 antibodies effectively and strongly detected DEFA5 in DV, UC, CC, IC tissue protein extracts (Fig. 4A, B), whereas the commercially available anti-DEFA5 antibody sc-53997 could detect DEFA5 faint bands in CC and IC tissue lysates only (Fig. 4C) and R&D#972207 antibody more specifically recognized DEFA5 particularly CC and IC tissue lysates (Fig. 4D). This suggests that this mAb only effectively detected DEFA5 when the protein was expressed at relatively high levels, but not at relatively low levels, as in DV and UC samples, even upon longer exposure.

### Immunoprecipitation analysis

**Figure 5:**
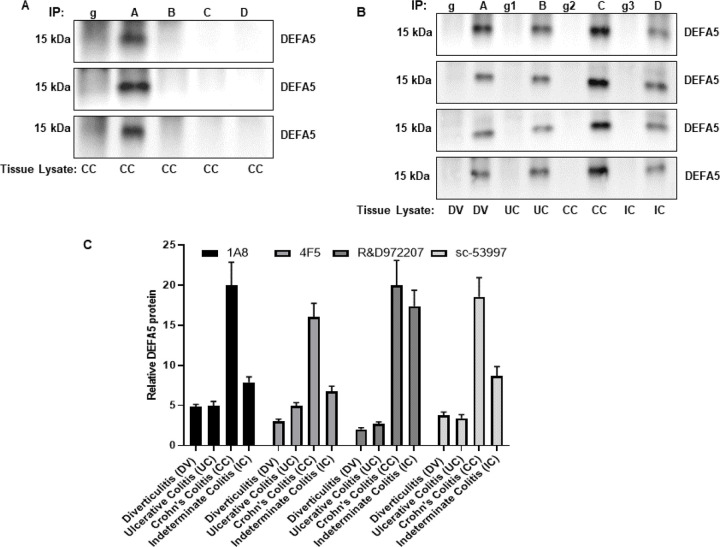
Specificity of antibodies for the capture of endogenous DEFA5 in complex proteins extracts and detection of DEFA5 in control and IBD patient tissue lysates by immunoprecipitation and western blotting. **(A)** 1 mg tissue extracts prepared from Crohn’s colitis (CC) sample for immunoprecipitation using mouse IgG control antibody, the two commercially available mouse monoclonal DEFA5 antibodies sc-53997, R&D972204, and newly developed mouse monoclonal DEFA5 antibodies 1A8, and 4F5. The immune complexes were probed with 1A8 (upper panel), 4F5 (middle panel) and R&D972207 antibodies (lower panel). A, B, C, D=IP with sc-53997 mAb against DEFA5 from CC. g=IP with normal mouse IgG. **(B)** The commercially available Mouse monoclonal DEFA5 antibody sc-53997 was used to immunoprecipitate DEFA5 protein from DV, UC, CC and IC tissue lysates and the 1A8, 4F5, R&D972207 and sc-53997 antibodies were used for standard Western blot analysis. A, B, C, D=IP with sc-53997 mAb against DEFA5 from DV, UC, CC and IC tissue lysates respectively. g, g1, g2, g3 =IP with normal mouse IgG. Immunoprecipitates were used in Western blot and each blot was incubated with 1A8 (first panel), 4F5 (second panel), R&D972207 (third panel) and sc-53997 (fourth panel) antibodies. **(C)**Densitometric analysis of the precipitated DEFA5 protein by ImageJ. Bars represent the mean intensity of the DEFA5 band from two patient samples relative to the corresponding IgG control.

Next, we tested the functionality of the antibodies during immunoprecipitation of tissue lysates prepared from active CC samples to identify the identification of DEFA5 capture antibodies. Commercially available sc-53997, R&D972204, and in-house 1A8 and 4F5 antibodies were used to immunoprecipitate DEFA5. The presence of DEFA5 in the immunoprecipitates was detected by immunoblotting using mouse monoclonal 1A8, 4F5, and 972207 antibodies for the immunoprecipitates with sc-53997, R&D Systems 972204, 1A8, and 4F5 antibodies. Only Santa Cruz antibody sc-53997 immunoprecipitated endogenous DEFA5 (Fig. 5A–C) establishing the most robust enrichment of the protein compared to the starting material. Antibodies R&D972204, 1A8, and 4F5 showed no DEFA5 immunoprecipitation, but DEFA5 in the immune complexes was strongly detected by 1A8 (Fig. 5A, upper panel), 4F5 (Fig. 5A, middle panel), and R&D972207 antibodies (Fig. 5A, lower panel) suggesting that the sc-53997 antibody was a preferred candidate for the capture of DEFA5 in tissue lysates, whereas 1A8, 4F5, and R&D972207 were the most effective antibodies for the detection of DEFA5 in immunoblots. Next, we assessed the degree to which 1A8, 4F5, and 972207 antibodies were selected for DEFA5 in immunoprecipitation assays. Immunoprecipitation studies were performed with the sc-53997 antibody as the capture antibody using UC, CC, IC, and control DV tissue lysates, followed by western blot analysis of the immunoprecipitated samples. The intensity of the bands provided information on the relative abundance of the protein in the control and IBD patient tissues, as detected by 1A8 (Fig. 5B, first panel), 4F5 (Fig. 5B, second panel), and R&D972207 (Fig. 5B, third panel). We also used the same sc-53997 antibody for both capture and detection in immunoprecipitation; the sc-53997 antibody, when occupying the binding site, was still capable of recognizing the remaining epitope on captured DEFA5 (Fig. 5B, fourth panel). The protein bands were quantified using ImageJ, revealing that all the detection antibodies effectively recognized DEFA5. However, the strongest DEFA5 signal was detected in CC and the weakest signals was DEFA5 in DV and UC (Fig. 5C). As expected, for the IC tissue used, the DEFA5 signal was stronger than that in DV and UC but weaker than that in IC. The semi-quantitative assay also suggests that the new antibodies, particularly 1A8, are the most sensitive at detecting CC compared to the other antibodies.

### Immunoprecipitation analysis to verify specificity of antibodies

**Figure 6:**
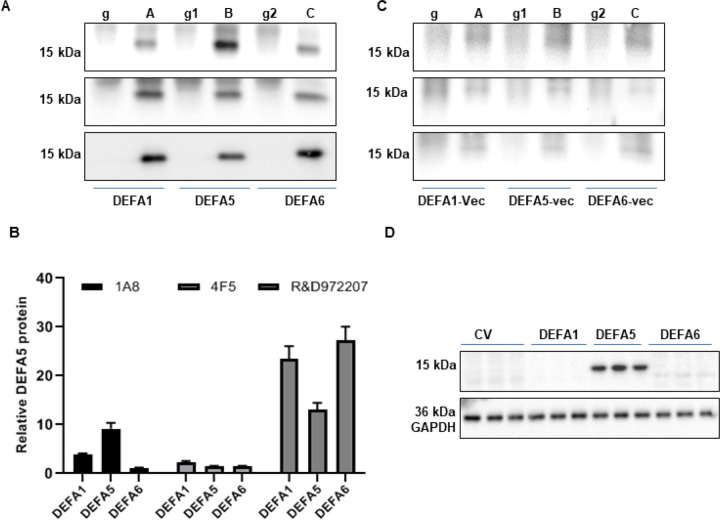
Specificity of the 1A8, 4F5and R&D972207antibodies for DEFA5. **(A)** Cell lysates from HEK293T cells expressing recombinant DEFA1, DEFA5 and DEFA6 α defensin family members were analyzed by immunoprecipitation and western blotting. (A) A, B, C=IP with sc-53997 mouse monoclonal antibody against recombinant DEFA1, DEFA5 and DEFA6 HEK293T cells lysates. g, g1, g2=IP with normal mouse IgG. Immunoprecipitates were used in Western blot and each blot was incubated with 1A8 antibody (upper panel) (A=recombinant HEK293T lysate, upper panel and control HEK293T lysate, lower panel), 4F5 antibody (middle panel) (B=recombinant HEK293T lysate, upper panel and control HEK293T lysate, lower panel) and R&D972207 antibody (lower panel). (C= recombinant HEK293T lysate, upper panel and control HEK293T lysate, lower panel). **(B)**Densitometric analysis of the precipitated DEFA5 protein. Bars represent the mean intensity of the DEFA5 band from two independent experiments relative to the corresponding IgG control. **(C)** A, B, C=IP with sc-53997 mouse monoclonal antibody against DEFA1-vector, DEFA5-vector and DEFA6-vector HEK293T cell lysates. g, g1, g2=IP with normal mouse IgG. Immunoprecipitates were used in Western blot and each blot was incubated with 1A8 (Figure 5C, upper panel), 4F5 (Figure 6C, middle panel) and R&D972207 (Figure 6C, lower panel) antibodies. **(D)** Cell lysates from HEK293T cells expressing recombinant DEFA1, DEFA5 and DEFA6 α defensin family member were analyzed by standard Western blotting. Detection of GAPDH was used as the loading control.

The specificity of antibodies 1A8, 4F5, and R&D972207 for DEFA5 was verified in cell lysates of transiently transfected HEK293T cells with cDNA encoding DEFA1, DEFA5, DEFA6, or negative control consisting of cells transfected with cDNA encoding the vector using only immunoprecipitation and western blotting. The 1A8 anti-DEFA5 monoclonal antibody was the most efficient in detecting a strong 15 kDa band in the HEK293T-DEFA5 cell extracts, which was weak or negligible in the HEK-293T-DEFA1 and HEK293T-DEFA6 cell lysates, thus verifying the specificity of 1A8 to DEFA5 and exhibited minimal non-confounding cross-reactivity with DEFA6 (Fig. 6A, upper panel). On the other hand, clone 4F5 anti-DEFA5 monoclonal antibody recognized 15 kDa bands in all three α-defensin recombinant proteins with relatively more staining in DEFA1 (Fig. 6A, middle panel), but the 4F5 antibody was not able to recognize recombinant DEFA5 similarly to endogenous, which may be because protein complexes may still be intact in the analyte, leading to masking of the antibody epitope. However, further studies are required to verify the specificity of 4F5. Whereas anti DEFA5 R&D972207 antibody displayed obvious cross-reactivity with DEFA1 and DEFA6 in the immunoprecipitation analysis. (Fig. 6A lower panel). No band was detected for mouse IgG, which was used as a negative control. Protein bands quantified using ImageJ showed that the 1A8 antibody recognized DEFA5 with a high degree of specificity, followed by the 4F5 antibody that showed higher reactivity with DEFA1, and the R&D972207 antibody had substantial reactivity with DEFA1, DEFA5, and DEFA6 (Fig. 6B), indicating that clone 1A8 and 4F5 anti DEFA5 antibodies are valuable tools for identifying DEFA5 to authenticate IC patients into UC or CC. No band was detected in the control lysates of HEK293T cells transfected with the expression vectors when 1A8 (Fig. 6C, upper panel), 4F5 (Fig. 6C, middle panel), and R&D972207 antibodies were used (Fig. 6C, lower panel). The specificity of the R&D972207 antibody for recombinant DEFA5 was also verified by western blotting of HEK293T cell lysates transiently expressing DEFA1, DEFA5, and DEFA6, or control cells expressing the vector only. The R&D972207 antibody detected a single band at 15 kDa in cell lysates containing DEFA5, but not DEFA1 or DEFA6, suggesting that R&D 927707 is very specific for DEFA5 recombinant protein and does not cross-react with DEFA1 or DEFA6 (Fig. 6D). Although this antibody was able to detect DEFA5 when highly expressed in HEK 293T cells, it was unable to detect lower levels of endogenous DEFA5 protein when expressed in DV and UC tissues, indicating a lack of specificity for DEFA5, as shown in Fig. 4D. In contrast, no band was observed in the cells transfected with the vector used as a negative control.

### Immunohistochemistry to verify specificity of antibodies

**Figure 7:**
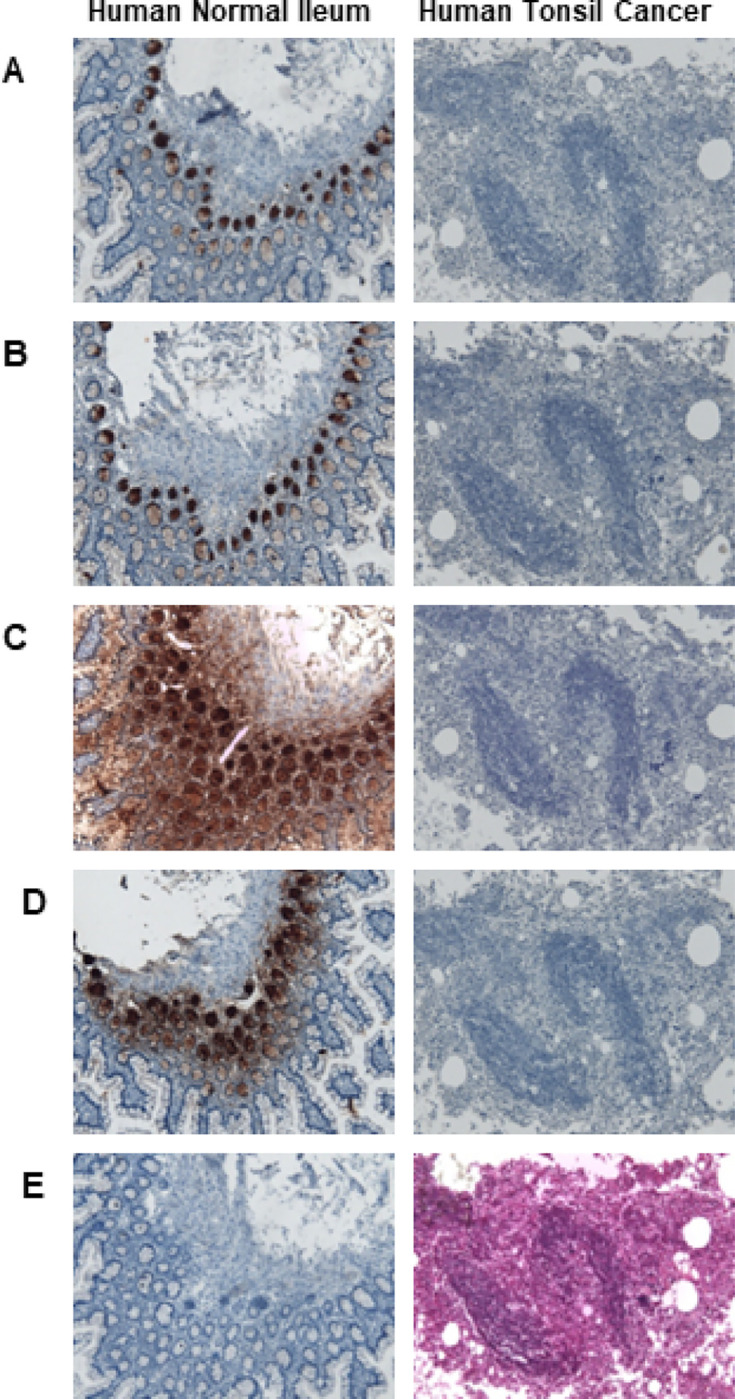
Immunohistochemical staining of formalin-fixed paraffin sections of human normal terminal ileum and human tonsil cancer tissue with 1A8 (panel A), 4F5 (panel B), R&D972207 (panel C) and sc-53997 (panel D) antibodies. Ileal section stained without primary DEFA5 antibody and stained only with HRP conjugated secondary antibody as control (panel E, left image) and tonsil tumor tissue section stained with H&E (panel E, right image) polymorphonuclear neutrophils can be seen.

We further tested the antibody staining of human tonsil tumor tissue sections by comparing them with human normal terminal ileal sections. The DEFA5 specificity of the 1A8 antibody (Fig. 7A, left image), 4F5 (Fig. 7B, left image), R&D972207 (Fig. 7C, left image), and sc-53997 (Fig. 7D, left image) were verified in sections of ileal tissues, where the antibodies strongly stained Paneth cells expressing DEFA5. Interestingly, the specificity of all four antibodies for paneth-cell-specific DEFA5 was demonstrated by the absence of HNP-1, HNP-2, HNP-3, and HNP-4 immunoreactive cells in consecutive sections of tonsil tumor tissue (Fig. 7A–D, right images), confirming the specificity of antibodies to DEFA5 in immunohistochemistry analysis. H&E staining of the tonsil tumor tissue revealed that the cells were morphologically identical to neutrophils (Fig. 7E, right panel).

### Clones 1A8 and 4F5 in ELISA assays

**Figure 8:**
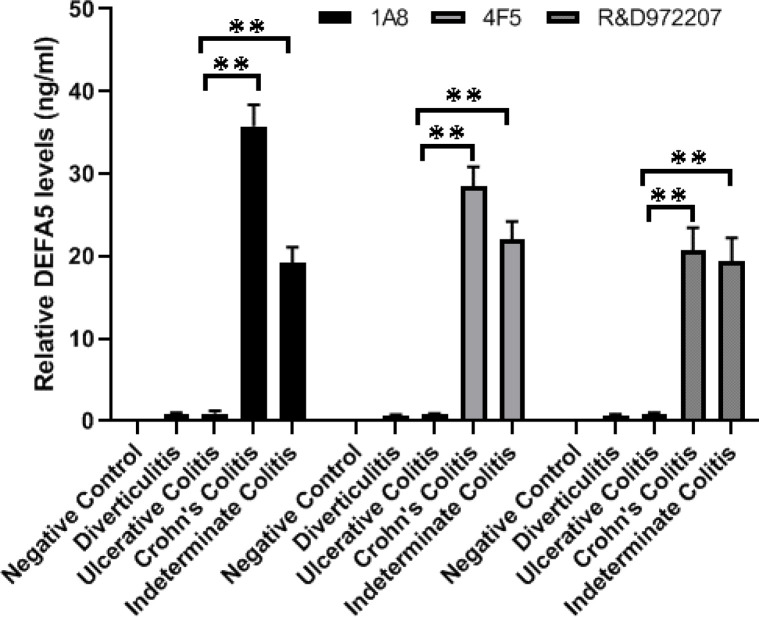
Determination of DEFA5 levels in control and IBD tissues samples by Sandwich ELISA. The commercially available sc-53997 mouse monoclonal antibody was used as the capture antibody and our in-house biotinylated mouse monoclonal 1A8, 4F5 and the commercially available R&D972207 antibodies were used as the detection antibodies. Antigens for the sandwich ELISA were patient tissue lysates from Diverticulitis (DV), Ulcerative colitis (UC), Crohn’s colitis (CC) and Indeterminate colitis (IC) patient samples and vector only lacking DEFA5 (EV). Bars represent mean ± S.D (n=3) values in ng/ml for the indicated antibodies. The statistical difference between UC and CC (1A8; *P*=0.0017, **P<0.01) or IC (*P*=0.0029, **P<0.01), UC and CC (4F5; *P*=0.0023, **P<0.01) or IC (*P*=0.0034, **P<0.01) and UC and CC (R&D972207; p=0.0063, **P<0.01) or IC (*P*=0.0078, **P<0.01) were significantly correlated.

Next, we analyzed the binding capabilities of 1A8, 4F5, and R&D972207 to endogenous DEFA5 protein using Sandwich ELISA. When binding was detected using biotinylated antibodies, clones 1A8 and 4F5 displayed a stronger binding profile than the R&D972207 antibody did. A standard curve constructed with the human recombinant α defensin 5 (DEFA5) standards (0.62 –40 ng/ml) showed that the OD_650_ values were directly correlated to the diluted standard concentrations. A negative control sample (EV), a vector lacking α-defensin 5, was used to check for non-specific binding and false-positive results. Blank samples were used to subtract the background from readings. The amount of DEFA5 protein in the DV, UC, CC, and IC samples was determined using a standard curve drawn using CurveExpert 1.4 Software. The DEFA5 levels in the DV, UC, CC, IC samples were 0.86 ng/ml (DV), 0.95 ng/ml (UC), 35.75 ng/ml (CC), 19.22 ng/ml (IC) detected with 1A8 antibody; 0.7 ng/ml (DV), 0.83 ng/ml (UC), 28.51 ng/ml (CC), 22.04 ng/ml (IC) with 4F5 antibody; and 0.71 ng/ml (DV), 0.89 ng/ml (UC), 20.68 ng/ml (CC), 19.36 ng/ml (IC) with R&D 972207 antibody (Fig. 8). This suggests that CC and IC samples led to a 20–34.9 -fold increase compare to DV and UC samples, demonstrating a significant difference between UC and CC or IC (1A8; *P* < 0.01; 4F5; *P* < 0.01; R&D; *P* < 0.01) (Fig. 8). This analysis suggests that DEFA5 levels < 1 ng/ml may represent DV or UC, whereas DEFA5 levels > 20 ng/ml may denote CC. It is also possible to conclude that the IC sample used in this assay was most likely from a patient with CC, which emphasizes the strength of this discovery.

## Discussion

To address IBD diagnostic challenges in clinical settings, we identified human alpha-defensin 5 (**DEFA5**, also abbreviated as **HD5**) restricted to areas of the colonic mucosa with aberrant expression of apparent crypt cell-like cells (CCLCs), which identifies an area of ectopic colonic ileal metaplasia that correlates with and is consistent with the diagnosis of CC [7, 8]. As a biomarker, DEFA5 can discriminate UC from CC by rectifying the IC cohort into authentic UC or CC. The scientific premise that *DEFA5* is found in CC and not in UC is not supported by the literature because *DEFA5* source is the ileum, which is reduced in Crohn’s ileitis [26–29]. The reverse is true in CC, DEFA5 is significantly 118-fold increased [7, 8]. In this light, *DEFA5* immunoreactivity in colonic endoscopy biopsies could be a potential diagnostic tool to accurately diagnose CC and provide the basis for resolving the ambiguity in the diagnosis of IBD to circumvent diagnostic delay and permit timely and accurate diagnosis and prescription of appropriate treatment options, especially surgically.

Many commercially available antibodies do not satisfy the criteria for specificity and affinity for DEFA5. The opportunity to study more than one antibody that recognizes different epitopes within the same protein using immunoprecipitation and ELISA is an excellent approach for identifying the most specific antibody for research and clinical applications. In the present study, we generated two in-house novel mAbs, 1A8 and 4F5, using hybridoma technology that is specific for DEFA5 and tested positive in a wide variety of immunoassays including western blotting, immunohistochemistry, immunoprecipitation, and ELISA. These results validate the high specificity of our mAbs against DEFA5. A general straight forward approach knockout validation (KO) to validate antibody specificity is to use CRISPR-Cas9 or RNA interference techniques to knockdown of the target gene in the human cell lines and compare the staining of the antibody by Western blotting or immunoprecipitation through cell lysates analysis before and after knockdown of the target gene [30–33]. It is difficult to use knockout validation to confirm the specificity of an antibody because the knockout cell not only causes the complete loss of the expression of the target protein but also decreases the expression of other family members containing highly homologous sequences that may cross-react with the antibody. Therefore, a negative control does not always mean that the antibody will not react with any other protein, except for the target protein. However, genetic strategies cannot be used for the validation of antibodies in human tissue samples, in which such knockout control is not possible, limiting this validation method for immunohistochemistry applications. We used a recombinant expression strategy to overexpress DEFA1, DEFA5, and DEFA6, which are not expressed in HEK293T cell lines used in the standard test methods. The reactivity of clones 1A8 and 4F5 was evaluated by immunoprecipitation prior to western blotting by analyzing cell lysates with and without expression vectors. The 1A8 antibody showed a strong band in HEK293T cells with DEFA5 recombinant expression and the fainter band with recombinant DEFA1 or DEFA6 verified its specificity. The 4F5 antibody was unable to detect the recombinant protein with a sensitivity similar to that of the endogenous protein, making it difficult to compare the specificity data to verify the reactivity with off-target DEFA1 and DEFA6 proteins. Further validation is necessary.

We successfully developed a Sandwich ELISA based on 1A8 and 4F5 as biotin-conjugated mAbs, which were used separately as detection antibodies, and sc-53997 was used as the capture antibody. Three independent standard curves were constructed at different time points, demonstrating that the system was reliable and reproducible. The limit of detection (LOD) for this assay was as low as 1.07 ng/ml, indicating a high sensitivity. The highest OD_650_ ratio for the positive and negative controls was obtained using 1A8 and sc-53997, establishing a high sensitivity and specificity for identifying and quantifying DEFA5 in IBD tissue lysates. In addition, we measured the expression of DEFA5 in active DV, UC, CC, and IC patient tissue samples, and found that DEFA5 levels in CC were significantly higher than those in UC and DV.

## Conclusion

The mAbs developed in this study were highly specific to the DEFA5 fragment, and the antibody pairs used in the ELISA facilitated the recognition of DEFA5 with a secure affinity. The 1A8 and 4F5 clones were successfully prepared for WB, IP, and ELISA, and the results of our study show that DEFA5 as a tissue biomarker has a promising future in the diagnosis of UC, from CC or IC to authentic UC, CC, or colitis with different pathological characteristics, and lay the foundation for subsequent clinical applications.

## Data Availability

The data supporting the findings of this study are included in this article. The names of the repository/repositories and accession numbers (s) can be found in the figures/supplementary material. Further inquiries can be made by the corresponding authors.

## Abbreviations

IBD: Inflammatory Bowel Disease
DV: Diverticulitis
UC: Ulcerative colitis
CC: Crohn’s colitis
RPC-IPAA: Restorative proctocolectomy with ileal pouch-anal anastomosis
IC: Indeterminate colitis
DEFA5: alpha defensin 5
PCLCs: Paneth cells-like cells
CCLCs: Crypt cells-like cells
HNP: Human Neutrophils Peptide
HEK293T: Human Embryonic Kidney 293 cells
Abs: Antibodies
mAbs: Monoclonal antibodies
